# Framework for Vehicle Make and Model Recognition—A New Large-Scale Dataset and an Efficient Two-Branch–Two-Stage Deep Learning Architecture

**DOI:** 10.3390/s22218439

**Published:** 2022-11-02

**Authors:** Yangxintong Lyu, Ionut Schiopu, Bruno Cornelis, Adrian Munteanu

**Affiliations:** 1 Department of Electronics and Informatics, Vrije Universiteit Brussel, 1050 Brussels, Belgium; 2 Macq S.A./N.V., 1140 Brussels, Belgium

**Keywords:** deep-learning, Vehicle Make and Model Recognition, two-branch strategy, large-scale vehicle dataset, Intelligent Transportation System (ITS)

## Abstract

In recent years, Vehicle Make and Model Recognition (VMMR) has attracted a lot of attention as it plays a crucial role in Intelligent Transportation Systems (ITS). Accurate and efficient VMMR systems are required in real-world applications including intelligent surveillance and autonomous driving. The paper introduces a new large-scale dataset and a novel deep learning paradigm for VMMR. A new large-scale dataset dubbed Diverse large-scale VMM (DVMM) is proposed collecting image-samples with the most popular vehicle brands operating in Europe. A novel VMMR framework is proposed which follows a two-branch architecture performing make and model recognition respectively. A two-stage training procedure and a novel decision module are proposed to process the make and model predictions and compute the final model prediction. In addition, a novel metric based on the true positive rate is proposed to compare classification confusion of the proposed 2B–2S and the baseline methods. A complex experimental validation is carried out, demonstrating the generality, diversity, and practicality of the proposed DVMM dataset. The experimental results show that the proposed framework provides 93.95% accuracy over the more diverse DVMM dataset and 95.85% accuracy over traditional VMMR datasets. The proposed two-branch approach outperforms the conventional one-branch approach for VMMR over small-, medium-, and large-scale datasets by providing lower vehicle model confusion and reduced inter-make ambiguity. The paper demonstrates the advantages of the proposed two-branch VMMR paradigm in terms of robustness and lower confusion relative to single-branch designs.

## 1. Introduction

Vehicle Make and Model Recognition (VMMR) or vehicle identification is the problem of identifying the vehicle’s make and model, i.e., the name of the vehicle manufacturer and the product, respectively, when given an input image/video that contains the vehicle. In recent years, VMMR gradually became an important part of Intelligent Transportation Systems (ITS) and is intensively studied by the research community.

Make and model recognition provides important information in many applications such as traffic management, intelligent surveillance, traffic behavior analysis, and traffic monitoring [1]. For example, the VMMR technology is employed in the traffic cameras installed in toll stations for the automatic detection and recognition of passing vehicles. In traffic monitoring, the vehicle model recognition is used by ITS to build the vehicle flow statistics used for the analysing of real-time traffic conditions. In traffic security [2], the make and model recognition can help locate stolen vehicles [3].

Make-and-model recognition is a very challenging problem due to the inherent visual properties of the vehicles such as [4]: (i) multiplicity, i.e., several vehicles of the same model can vary both in terms of color and captured poses; (ii) inter-make ambiguity, by which vehicles from different makes can have similar appearances; (iii) intra-make ambiguity, that is vehicles from different models and the same make can have similar appearances. To alleviate these issues, alternative approaches to identify vehicle make and model, such as manual observation and Automated License Plate Recognition (ALPR) [5,6,7,8], are proposed. However, these approaches have some limitations. Manual observation requires the observer to pay an increased attention to details and to have a good knowledge of the diverse set of vehicle makes and models available in different colors. Moreover, this approach is impractical in applications that require automatic VMMR methods. When employing ALPR algorithms, the license plate and vehicle make and model information might not always be legally available. Moreover, fake license plates are still present on the roads and highways; in addition, one can use the detected make and model to verify if they correspond indeed to the detected number plate and trigger alarms in the case of mismatches.

One of the approaches proposed by the research community is to employ traditional methods [9,10,11,12] which rely on Scale-Invariant Feature Transform (SIFT) [13] and Speeded Up Robust Features (SURF) [14] to identify vehicle makes and models. However, these methods suffer from limitations as the input images must be captured from a fixed viewpoint, e.g., frontal view, such that the camera sensor is able to capture specific features of the vehicle, e.g., the logo and frontal lights.

With the development of deep-learning techniques for VMMR, the application of several well-known backbones [15,16,17] in solutions for fine-grained vehicle classification was proposed [1,4,18]. However, none of these methods focuses on the reducing vehicle model classification confusion problem. In this paper, we propose a deep-learning-based approach for VMMR which reduces the classification ambiguity by employing two independent classifiers that perform coarse-grain make recognition and fine-grain model recognition respectively. The main contributions of the paper are summarized as follows:

(a) A new Diverse large-scale VMMR (DVMM) dataset is introduced, which contains images with the most popular 23 vehicle makes and 326 vehicle models available in the European automotive market.
(b) A novel Two-Branch–Two-Stages (2B–2S) framework is proposed. The architecture contains one branch performing vehicle make recognition and another branch to perform vehicle model recognition, whereby the make recognition branch is introduced to reduce classification confusion. The network model is trained using a two-stage procedure, whereby each recognizer branch is trained separately to compute the initial prediction. A novel decision module is proposed to further process the make and model predictions and compute the final model prediction.
(c) A novel metric, called Gain (G) score, is proposed based on the true positive rate and employed to measure the ability to decrease classification confusion of the proposed 2B–2S framework relative to the corresponding one-branch model recognition method.
(d) A complex experimental evaluation carried out on traditional VMMR datasets, such as Compcar [1] and VMMRDB [4], and the new DVMM dataset demonstrates the potential offered by the two-branch processing paradigm. Experimental results show that the proposed 2B–2S significantly reduces the inter-make ambiguity and provides a lower vehicle model confusion than the corresponding one-branch model recogniser, for different backbone designs.

The remainder of this paper is organized as follows. Section 2 describes the traditional datasets used for VMMR and outlines the state-of-the-art methods. Section 3 introduces the new DVMM dataset. Section 4 describes the proposed 2B–2S framework. Section 5 presents the experimental results and performance analysis of the proposed VMMR framework. Section 6 discusses future work directions. Section 7 draws the conclusions of this work.

## 2. Literature Review

### 2.1. Existing VMMR Datasets

In recent years, VMMR starts to attract an increased attention in the research community. However, its development is slower compared to other directions in computer vision, such as image classification, object detection, and instance segmentation, to name a few. One reason is the lack of diverse large-scale datasets for training deep neural networks. One notes that the manual annotation of high-quality VMMR datasets not only requires a lot of manpower, but also that the annotation team must have the necessary training and skills to differentiate between the different vehicle models available in the dataset.

The existing VMMR datasets can be divided into two categories: (i) web-scraping datasets; and (ii) surveillance datasets. The web-scraping datasets are mainly extracted from websites using web scraping (e.g., by analyzing the second-hand vehicle trading websites), which can be done manually or by employing an automated process. The web-scraping datasets can be semi-automatically annotated based on the text description provided by the users on the found websites. The dataset image samples are captured from various viewpoints using various camera devices and viewing conditions. The surveillance style datasets are mainly acquired by the surveillance cameras, which are configured to capture either the vehicle’s side–view [19] or top-front–view [20,21]. In this case, in contrast to web-scraping datasets, the vehicle textual description is not available, therefore, most surveillance VMMR datasets are manually annotated or indirectly annotated by employing a license plate recognition algorithm. One can easily note the limitation of the surveillance VMMR datasets, as only the vehicle’s side or top-front views are captured from fixed viewpoints above the road or highway.

The existing web-scraping VMMR datasets can be further divided based on their size into three categories: (i) small-scale, containing less than 10,000 samples; (ii) medium-scale, containing between 10,000 and 150,000 samples; and (iii) large-scale, containing more than 150,000 samples. In [22], a web-scraping dataset which contains 7570 samples is introduced. The vehicles are manually divided into seven classes based on their body-style, e.g., Sport Utility Vehicle (SUV), crossovers, hatchbacks, wagons, sedan, coupes, and others. However, such datasets can be used mostly in vehicle body recognition applications rather than make-and-model recognition applications.

The VMMR datasets introduced in [1,18,23,24] belong to the medium-scale category. The Compcar dataset [1] contains both web-scraping and surveillance data. The web-scraping part of the dataset contains 52,083 samples annotated using 431 vehicle models. The Frontal-103 dataset [18] contains 65,433 frontal–view samples annotated using 1759 models and 103 makes. The Stanford car dataset [23] contains 16,185 samples manually annotated using 196 vehicle models. The 43,615 samples from the FZU car dataset [24] were manually annotated using 297 vehicle models. These medium-scale datasets contain various vehicle models, however, the number of samples of each vehicle model is limited. For example, most of the classes in the Frontal-103 dataset [18] contain less than 50 samples. A similar problem can be observed for the large-scale VMMR dataset [4], called VMMRDB, which is the first large-scale dataset collected for VMMR and automatically annotated using the textual description found on websites. VMMRDB contains 291,752 samples covering 9710 vehicle models available in the American automotive market. However, most of the vehicle models contain less than 100 samples. Due to its high relevance to our work, the experimental validation presented in Section 5 is also carried out on the VMMRDB dataset.

### 2.2. State-of-the-Art Methods

In general, the state-of-the-art methods proposed in the VMMR research area can be divided into two categories: (i) traditional feature-based VMMR solutions; and (ii) Convolutional Neural Network (CNN) based VMMR solutions designed using innovative Machine Learning techniques.

The traditional feature-based solutions are usually designed based on the following general framework. A certain region of interest (ROI) of the vehicle is first selected as input. For each ROI, an extraction algorithm is applied to extract certain features, which are then represented in a global manner. Subsequently, classification algorithms, such as Support Vector Machines (SVM), are then employed to process the extracted global features and compute the final make and model predictions. The algorithms proposed in [10,11,12] take as input a sample-image containing the frontal view of a vehicle, from which an ROI input containing the vehicle’s windshield with or without its hood is extracted. The SIFT [13] or SURF [14] features are then employed to process the ROI and extract specific features. The extracted features are then embedded into a global representation using a bag-of-features strategy. The global representations are finally multi-classified by the SVM algorithm. The methods proposed in [25,26] also employ a feature-based pipeline, but follow a two-stage approach. In [25], the authors propose to first classify some easy cases which have a high score, while the other difficult cases are classified in the second stage. A similar algorithm is proposed in [26], where two more classes are introduced in the second stage for the unknown vehicles. One can note that traditional feature-based methods are limited to input images which capture only the frontal view of the vehicles. These methods provide a good performance on the datasets which contain a limited number of vehicle makes and models; however, when dealing with large-scale datasets, their performance does not always meet the expectations.

In the Machine Learning domain, the research community has proven that deep-learning-based solutions provide remarkable gains over the traditional feature-based solutions for many computer vision tasks, such as image classification, object detection, object recognition, and many more. Therefore, in recent years, many research groups have focused on designing novel CNN-based solutions for the VMMR problem. In [4,18,27,28], the proposed CNN-based solutions follow an approach, by which existing successful architectures, usually applied for image classification and commonly known as backbones, are employed to perform VMMR on a specific dataset. One can note that such well-known backbones must be modified such that the last dense layer computes the probability distribution over the classes of vehicle models found in the dataset.

The Visual Geometry Group (VGG) [29], AlexNet [15], ResNet [16], or DenseNet [17] backbones are employed for VMMR; however, they were designed for other computer vision tasks. In other approaches, an existing architecture and/or loss function are optimized for a specific task. In [30], the authors optimise the SqueezeNet backbone [31] in a residual manner. Bypass connections are introduced between the fire modules of the original SqueezeNet backbone to obtain a CNN-based solutions for real-time surveillance VMMR. The optimised architecture proposed in [22] employs AlexNet [15] but the algorithm is designed for vehicle body-style recognition, where only seven body type classes are used; this is an oversimplified problem compared to the VMMR problem tackled in this work. Moreover, unlike the existing CNN-based solutions, the proposed 2B–2S framework is designed to reduce the inter-make ambiguity, which is an important factor affecting the recognition accuracy in existing VMMR methods.

## 3. Proposed Large-Scale VMMR Dataset

A new large-scale VMMR dataset, termed DVMM and belonging to web-scraping category is introduced in this section. The proposed DVMM dataset collects images for the most popular 23 vehicle makes operating in the European automotive market. The image samples in this new are collected from several second-hand trading and vehicle review websites. Such vehicle review websites contain a large diversity of vehicle models, most of the images for a given model being captured from different viewpoints.

**Figure 1:**
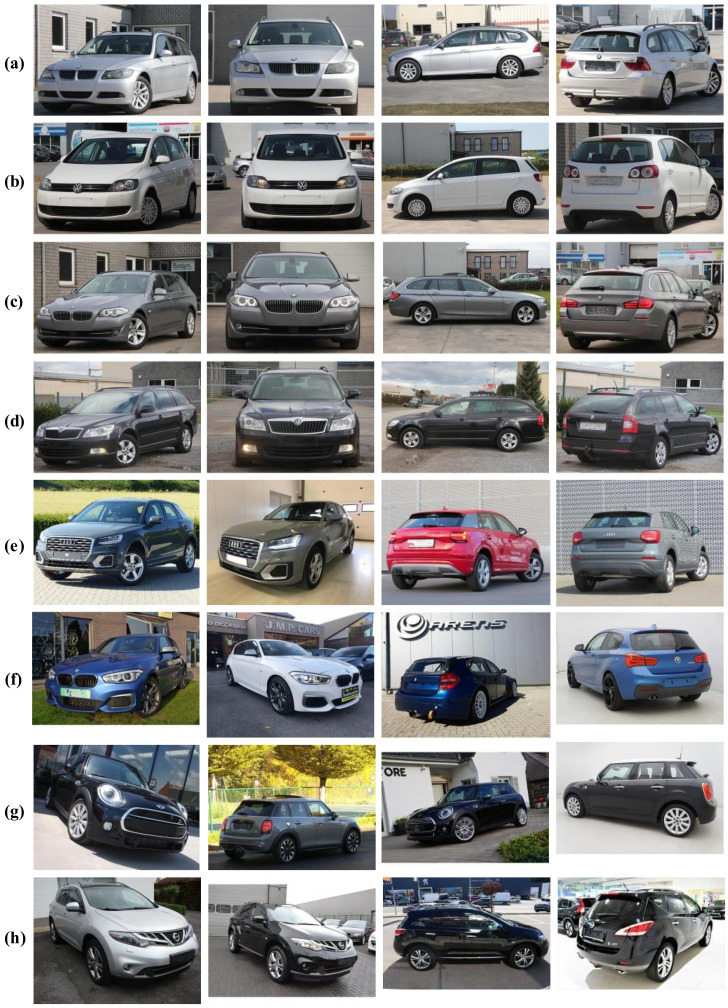
Sample images from the proposed DVMM dataset: (**a**) *BMW 3 Series*; (**b**) *Volkswagen Golf*; (**c**) *BMW 5 Series*; (**d**) *Skoda Octavia*; (**e**) *Audi Q2*; (**f**) *BMW 1 Series*; (**g**) *Mini Cooper*; (**h**) *Nissan Murano*.

Figure 1a–d show four different image samples of four different vehicle models taken from the front-side, front, side, and back viewpoint of the vehicle, respectively. One can note that the images extracted from the second-hand trading websites are more diverse, as most images are taken with different cameras, at different resolutions, and under various illumination conditions.

Figure 1e–h show 16 image samples representing four different model classes with different colors taken from different viewpoints and under various conditions (camera types, image resolutions, illumination conditions and background settings). One can note that this kind of diversity ensures a more realistic classification and enforces more generality.

In [1,4,18,27], the CNN-based VMMR solutions provide a vehicle classification at the make-model-year level. In this paper, in order to provide enough samples for each class, the new DVMM dataset contains information only at the make-and-model level.

A total number of 281,133 samples are initially collected from different websites. For most of the samples we assign a corresponding text label with the vehicle’s make and model using a semi-automatic annotation process. Since not all the interiors or zoomed-in parts of the cars provide interesting features to train a robust deep learning model for VMMR, some of the images are removed by running the well-known object detection algorithm, You Only Look Once (YOLO) [32]. After applying this cleaning process, the DVMM dataset contains 228,463 samples covering 326 different vehicle models, where each vehicle model contains more than 100 samples.

**Figure 2:**
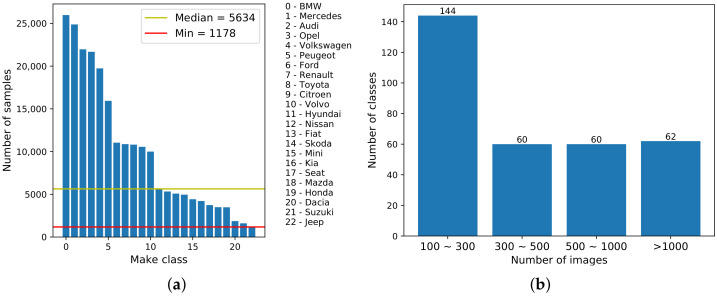
(**a**) Distribution of make classes in new DVMM dataset. The red line marks the minimum number of samples corresponding to 1178. The yellow line marks the median number of samples corresponding to 5634. The 23 vehicle make class labels are presented on the right side. (**b**) The four-bin histogram of the 326 vehicle model classes in DVMM.

Figure 2a shows the distribution of the DVMM classes when keeping the classification at a make level and presents the list of the 23 vehicle makes selected for the dataset. One can note that: (i) the *Jeep* make class contains the smallest number of image-samples (more exactly, 1178 samples); (ii) 6 out of 23 vehicle make classes contain more than 15,000 samples; and (iii) around 50% of the make classes contain more than 10,000 samples.

The top five makes having the largest amount of samples include *BMW*, *Mercedes*, *Audi*, *Opel*, and *Volkswagen*, which are the most popular makes available in the European automotive market.

Figure 2b shows the four-bin (100–300, 300–500, 500–1000, and >1000) histogram of the new DVMM dataset when keeping the classification at model level. One can note that all of the vehicle model classes contain more than 100 samples, and around 45% of vehicle model classes contain between 100 and 300 samples. There are around 60 vehicle model classes for each category which contain 300–500, 500–1000, or more than 1000 image samples. These figures indicate that the proposed DVMM is a large-scale and diverse dataset that can be used for training a vehicle make recogniser and/or a vehicle model recogniser. A comparison between the proposed dataset and the currently available VMMR datasets is provided in Section 5.1.

## 4. Research Methodology

Our experiments show that vehicle fine-grained classification at make level always provides a good performance when the VMMR neural network architecture is designed based on some well-known architectures, like the DenseNet201 backbone [17]. One also notes that a classification at make level is coarser-grained compared to a vehicle classification at model level. Moreover, classification confusion always occurs in case of appearance-alike vehicle models from different makes, that is, inter-make ambiguity.

Based on these observations, the basic ideas followed in the proposed CNN-based VMMR method are to:

(i) introduce in the network architecture an extra make recogniser branch with a good performance that can provide additional make information regarding the vehicle classification, and

(ii) design an extra refinement stage which makes use of the prediction computed by the make recognizer to improve the initial model prediction.

Section 4.1 presents the proposed Two-Branch–Two-Stage (2B–2S) framework for VMMR. Section 4.2 introduces a novel evaluation metric to compare two VMMR solutions based on different network designs. In this work, the novel metric is mainly used to compare the performance of the proposed 2B–2S framework with the corresponding one-branch model recogniser.

### 4.1. Proposed Architecture

**Figure 3:**
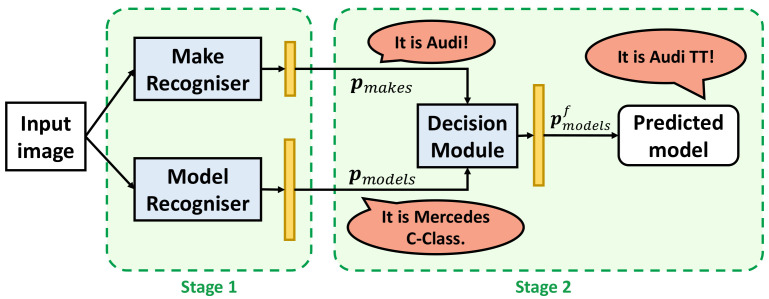
The scheme of the proposed Two-Branch–Two-Stage (2B–2S) framework for VMMR.

Figure 3 depicts the scheme of the proposed 2B–2S framework for VMMR. 2B–2S is a network design with two branches with two separate backbones and employs a two-stage training procedure. The first branch employs a vehicle make recogniser backbone to predict the vehicle’s make. The second branch employs a model recogniser backbone to predict the initial vehicle’s model. Each branch operates on the input RGB vehicle images and consists of a CNN-based feature extraction backbone. The number of output classes in the last fully-connected (*fc*) layer in each make and model backbone is set as the number of vehicle makes and vehicle models, respectively.

In the first training stage, each branch is trained separately at the make and model level, respectively, on the same dataset. In the second stage, the weights of the make recogniser and the model recogniser are fixed. The two outputs are then concatenated and taken as input for a novel module design to reduce classification confusion, called Decision Module (DM), as illustrated in Figure 3.

Table 1 presents the neural network configuration of the proposed DM module, which contains a sequence of four *fc* layers. The first three layers are equipped with a Rectified Linear Unit (ReLU) activation function, while the last *fc* layer is equipped with a *softmax* activation function so that the neural network architecture can predict the final vehicle model classification. The number of output classes in the sequence is set as 1024, 1024, 768, and the number of vehicle models (#Model), respectively. One can note that the layer configuration of the proposed DM module is set by taking into account the number of make and model classes found in general in VMMR datasets, and is designed to have a medium complexity, which is enough to refine the initial model prediction based on the make prediction.

Similar to [4,16,18], we use the cross entropy loss function, given by:

(1) $$L_{\theta }=-\frac{1}{N}\sum_{i=1}^{N}\left[y_{i}\log\hat{y_{i}}+\left(1-y_{i}\right)\log\left(1-\hat{y_{i}}\right)\right],$$

where $y_{i}$ is the true label and $\hat{y_{i}}$ denotes the corresponding prediction. *N* represents the number of data samples and θ∈{make,model} indicates the corresponding classifier. In the first stage, two independent loss functions, $L_{make}$ and $L_{model}$ are used to optimise each branch respectively, while the DM module is trained during the second stage by minimising $L_{model}$.

In our experiments, we propose to test three different backbone configurations for the model recogniser branch: (i) the AlexNet backbone [15], (ii) the ResNet50 backbone [16], and (iii) the DenseNet201 backbone [17].

The AlexNet architecture [15] is originally designed to perform image classification on the famous ImageNet dataset. It contains a sequence of five convolutional layers followed by a sequence of three *fc* layers. In [15], the authors highlight that the architecture’s depth play an important role in achieving a high performance for the classification task.

ResNet50 [16] contains a much deeper architecture depth. Its design represents an important milestone for solving the degradation problem when training deeper neural networks. The authors are the first to introduce the concept of deep residual learning. The ResNet50 architecture follows a similar approach as VGG [29], but introduces shortcut connections every few stacked layers. It is designed based on the following observations: (i) the number of filters is kept constant when processing feature maps having the same patch resolution; (ii) the number of filters is doubled when the resolution of the feature map is halved.

The DenseNet201 architecture [17] follows a more densely-connected approach, which connects each layer to every other layer in a feed-forward fashion. Both ResNet50 and DenseNet201 architectures achieve good performance when employed to provide a fine-grained classification. In this work, the backbone of the make recogniser only employs a DenseNet201 backbone as our experiments show that the DenseNet201-based make recogniser provides the best performance.

### 4.2. Proposed Evaluation Metric

In literature, the performance of the CNN-based VMMR methods is evaluated using the Accuracy (acc) and F1-score metrics, which are defined as follows:

(2) $$acc=\frac{\sum_{k=1}^{L}TP_{k}}{N},$$

(3) $$F1=\frac{\sum_{k=1}^{L}F1_{k}}{L},$$

where *L* is the number of classes, *N* is the number of samples, and $F1_{k}$ is the *F*1 score computed for the *k*-th class as follows:

(4) $$F1_{k}=\frac{TP_{k}}{TP_{k}+\frac{1}{2}\left(FP_{k}+FN_{k}\right)}.$$

TP, FP and FN are the true positive, false positive, and false negative values, respectively, which are computed for each class.

The traditional Acc and F1-score metrics are used to measure the performance of the VMMR solutions in this paper. Moreover, in order to evaluate the ability to reduce classification confusion, we propose to introduce a new metric, called Gain (G) score. Here, G-score is employed to compare the vehicle model confusion of the one-branch model recogniser, called 1B, against the proposed 2B–2S framework, when both employ the same backbone in the model recognizer branch.

Let us define the true positive set over all classes available in the dataset as C(A), given by:

(5) $$C\left(A\right)=\overset{L}{\underset{k=1}{⋃}}TP_{k}\left(A\right),$$

where $TP_{k}\left(A\right)$ denotes the true positive predictions of class *k* by using architecture *A* and A∈{1B,2B–2S}. To measure the confusion reduction ability, we compare the number of samples belonging to two sets: (1) P={x|x∈C(1B),andx∉C(2B–2S)}, and (2) Q={x|x∈C(2B–2S),andx∉C(1B)}. Let |·| define the cardinality of a set. If |P|<|Q|, 2B–2S decreases more the vehicle model confusion compared to 1B and vice versa. The G-score is defined using the relative percentage of *P* and *Q*, which is computed as:

(6) $$G\left(P\right)=\frac{\left|P\right|}{\left|P|+|Q\right|},\;G\left(Q\right)=\frac{\left|Q\right|}{\left|P|+|Q\right|}.$$

If we find that G(P)<G(Q), then the predictions of the proposed 2B–2S contain less classification confusions than 1B. Conversely, if G(P)>G(Q), 1B provides more convincing classifications than 2B–2S. An analogous definition can be made for the G-score when comparing the vehicle make confusion reduction of 1B and the corresponding 2B–2S.

In addition to the G-score, in this paper, the difference in performance between the proposed 2B–2S framework and the corresponding 1B approach is visualized using a Confusion Matrix (CM) based metric. The Binary Difference Confusion Matrix (BDCM) is introduced as follows:

(7) $$\begin{array}{c} \mathrm{BDCM}\left(i,j\right)\;=\;\left\{\begin{array}{cc} 0\;\; & \;\;\mathrm{if}\;CM_{2B-2S}\left(i,j\right)=CM_{1B}\left(i,j\right)\\ \delta \;\; & \;\;\mathrm{if}\;i=j\;\&\;CM_{2B-2S}\left(i,j\right)\neq CM_{1B}\left(i,j\right)\\ -\delta \;\; & \;\;\mathrm{if}\;i\neq j\;\&\;CM_{2B-2S}\left(i,j\right)\neq CM_{1B}\left(i,j\right) \end{array}\right. \end{array}$$

where δ is computed as follows:

(8) $$\delta =\frac{CM_{2B-2S}\left(i,j\right)-CM_{1B}\left(i,j\right)}{|CM_{2B-2S}\left(i,j\right)-CM_{1B}\left(i,j\right)|},$$

where *i* is the row index corresponding to the ground truth labels; *j* is the column index corresponding to the predicted labels; $CM_{1B}$ is the confusion matrix computed for 1B for vehicle model recognition; and finally $CM_{2B-2S}$ is the confusion matrix computed for 2B–2S for vehicle model recognition. Therefore, in Equation (7), when i=j, the ground truth label is the same as the predicted label; while, when i≠j, the ground truth label is different from the predicted label.

In Equation (8), one can note that we have δ∈{−1,1}. Therefore, the following cases are distinguished:

(i) if BDCM(i,j)=1, the proposed 2B–2S framework provides a better performance than 1B;
(ii) if BDCM(i,j)=0, the two methods have the same performance; and
(iii) if BDCM(i,j)=−1, the 1B method provides a better performance than 2B–2S.

In conclusion, if the BDCM matrix contains a larger number of ‘1’s than ‘−1’s without counting different vehicle models belonging to the same make, in general, the proposed 2B–2S framework provides a better performance than 1B and reduces the inter-make ambiguity.

## 5. Experimental Validation

The experimental validation is performed over traditional VMMR datasets and the new DVMM dataset. Section 5.1 presents the VMMR datasets used for training neural network models. Section 5.2 presents the experimental results and analyses the performance of the proposed 2B–2S framework. Section 5.3 presents the ablation study.

### 5.1. Training VMMR Datasets

The new DVMM dataset has a similar structure as the famous VMMRDB dataset [4]. A comparison between DVMM and VMMRDB is presented below to demonstrate the advantages of using a more diverse large-scale VMMR dataset for training neural network models.

The VMMRDB-3036 dataset [4] collects only those classes containing more than 20 vehicle image samples, and contains a total of 3036 vehicle models. For a fair comparison with DVMM, the VMMRDB-3036 dataset is rearranged at the make-and-model level classification rather than the make-model-year level classification used in [4].

In the following, let us denote by *S*-*L* the training VMMR dataset obtained by extracting *L* vehicle model classes from the VMMR dataset *S*. In this work, the image data in the original VMMRDB-3036 dataset [4] is rearranged from the make-model-year classification performed in [4] to a make-and-model classification addressed in this work; the re-arrangement corresponds to a dataset that contains 43 vehicle make classes and 495 vehicle model classes, being denoted here as VMMRDB-495. It is important to highlight that the image data in VMMRDB-495 is exactly the same as in the original VMMRDB-3036 dataset [4], the only difference is that the data is organized in a make-and-model set of classes and not in a make-model-year set of classes as in [4].

In this work, we also compare the VMMR solutions over the Top-100 most populated vehicle models and the Bottom-100 less populated vehicle models found in each VMMR dataset. To analyse the generality and applicability of the new DVMM dataset, we propose to employ on the model recogniser branch one of the following three backbones: AlexNet [15], ResNet50 [16], and DenseNet201 [17], where the last network layer in the backbone is replaced with a global average pooling layer and a *fc* layer equipped with a *softmax* activation function.

**Figure 4:**
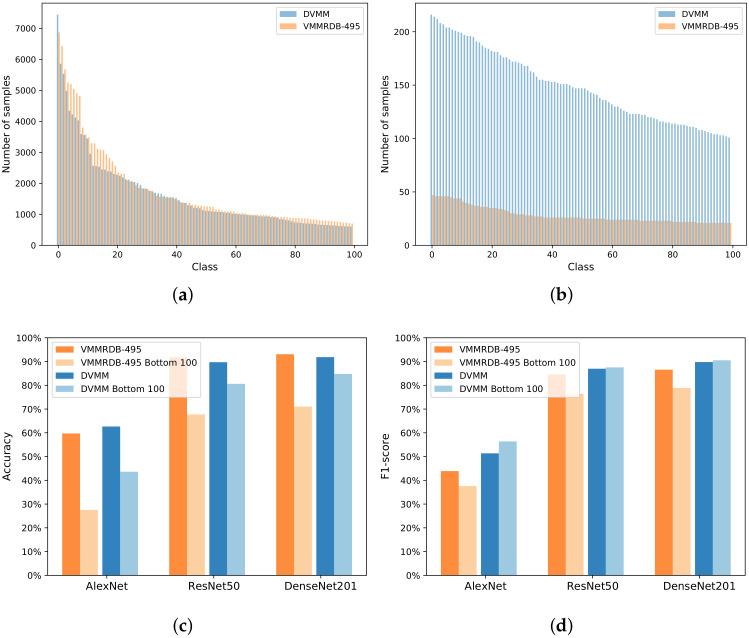
Distribution of model classes in the VMMRDB-495 [4] and DVMM datasets: (**a**) Top-100 classes (most populated); (**b**) Bottom-100 classes (least populated). Performance comparison for the 2B–2S framework, where the model recogniser branch is designed based on AlexNet [15], ResNet50 [16], or DenseNet201 [17] backbone: (**c**) Accuracy; (**d**) F1-score.

Figure 4a shows the distribution of the Top-100 vehicle model classes in the new DVMM dataset and the traditional VMMRDB-495 dataset. Figure 4b shows the distribution of the Bottom-100 vehicle model classes in the new DVMM dataset and the traditional VMMRDB-495 dataset. One can note that the DVMM–Top-100 and VMMRDB-495–Top-100 datasets have similar distributions. However, all of the vehicle model classes in the DVMM–Bottom-100 dataset contain more than 100 vehicle image samples, while all vehicle models in the VMMRDB-495–Bottom-100 dataset contain less than 50 vehicle image samples.

Figure 4c shows that for all three tested backbones (AlexNet, ResNet50, and DensNet201), when comparing the performance of the models trained using the full dataset and the corresponding Bottom-100 dataset, the new DVMM dataset has a much smaller drop in accuracy than the traditional VMMRDB-495 dataset. More exactly, the accuracy drop from DVMM (marked with blue) to DVMM–Bottom-100 (marked with light-blue) is much smaller than the accuracy drop from VMMRDB-495 (marked with orange) to VMMRDB-495–Bottom-100 (marked with light-orange). The performance drops over VMMRDB-495 are more than 20% for all three backbones, while the performance drops over DVMM are around 10% for AlexNet and less than 10% for both ResNet50 and DenseNet201.

Figure 4d shows that for all three backbones, the F1-score result drops with more than 5% over VMMRDB-495. While for all three backbones, the F1-score results are increasing over DVMM. One can note that the models trained using the VMMRDB-495 dataset are affected by under-fitting for the Bottom-100 vehicle models as these classes do not contain enough training samples. However, the models trained using the new DVMM dataset are not affected by under-fitting for the Bottom-100 as these classes contain more training samples.

### 5.2. Experimental Results

In this section, we analyse the performance of the proposed 2B–2S framework over small-, medium-, and large-scale datasets with or without constraining the number of available samples for the vehicle model class. Section 5.2.1 presents the experimental setup. Section 5.2.2 presents the numerical results and analysis of the proposed 2B–2S framework.

#### 5.2.1. Experimental Setup

Since most of the traditional VMMR datasets contain a limited number of largely populated vehicle model classes, in a first experiment, we propose to generate a set of small-scale VMMR training datasets, denoted *S*-51, where each dataset collects the most populated Top-51 vehicle model classes from one of the following dataset: Compcar [1], VMMRDB [4], and the new DVMM dataset; i.e., S∈{Compcar,VMMRDB,DVMM}. The neural network models are trained using the most representative classes from each dataset. The Compcar and VMMRDB dataset are rearranged to a make-and-model level classification.

In a second experiment, we propose to evaluate the performance of the proposed 2B–2S framework when the neural network models are trained using large-scale VMMR datasets (containing hundreds of model classes), such as the traditional VMMRDB-495 dataset and the new DVMM dataset.

The neural network models are trained by randomly splitting the datasets using a 70–30% ratio for Training–Testing. For a fair comparison with the new DVMM dataset, the vehicle models containing less than 100 image samples are also removed from the VMMRDB-495 Test set. Table 2 shows the specifications of all the VMMR datasets used in this paper to carry out experiments. One can note that Compcar-51 [1], VMMRDB-51 [4], and DVMM-51 are small- and medium-scale datasets, while VMMRDB-495 [4] and new DVMM are large-scale datasets.

The proposed 2B–2S framework is designed so that the make recogniser branch always employs a DenseNet201 backbone [17], while the model recogniser branch employs one of the AlexNet, ResNet50, or DenseNet201 backbones. The ResNet50 and DenseNet201 backbone are initialized using weights trained on the ImageNet dataset [33] for image classification, while AlexNet [15] is trained from scratch. The training image samples are resized to the 256×256 resolution and are augmented using flipping and $90^{\circ }$ rotation operations. The AlexNet backbone, the ResNet50 backbone, and the Decision Module are trained using a batch size of 32 samples. Due to the GPU memory limitation, the DenseNet201 backbone is trained using a batch size of 16 samples.

The proposed method is implemented in Tensorflow and optimized by Adam optimizer [34]. In Table 3, we list the detailed training schema of the model trained on DVMM. At Stage 1, all the learning rates (lr) are multiplied by the corresponding weight factor after epoch 20; the model learns with learning rates decayed after epoch 5 at Stage 2. The experiments are performed on a machine equipped with an NVIDIA GTX 2080 Ti GPU.

In this work, the performance of the following methods is compared: (i) the proposed 2B–2S framework; and (ii) the corresponding one-branch model recogniser, 1B. The VMMR results of each method are reported at the following classification level: (i) make; (ii) model. The accuracy, F1-score, *G*-score, and BDCM metrics are used to evaluate performance of the VMMR solutions.

#### 5.2.2. Numerical Results and Analysis

**Figure 5:**
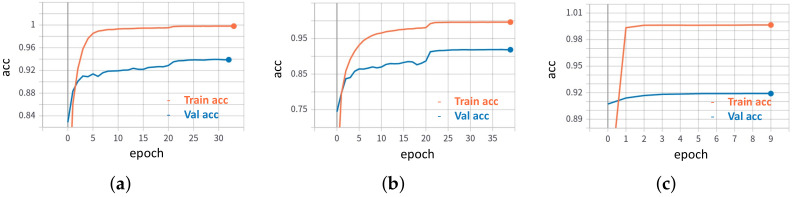
Accuracy plot during the training process of the proposed 2B–2S on the DVMM dataset. (**a**) Make recogniser (Stage 1). (**b**) Model recogniser (Stage 1). (**c**) The DM module (Stage 2).

In the proposed 2B–2S framework, the make recogniser branch always employs a DenseNet201 [17] backbone to assist the model recogniser branch as DenseNet201 [17] achieves the best make recognition results on the challenging datasets, as shown in Table 4. Table 5 shows the vehicle make recognition results for the 1B method when employing a DenseNet201 [17] backbone trained on: (i) three small- and medium-scale datasets, Compcar-51 [1], VMMRDB-51 [4], and new DVMM-51; and (ii) large-scale datasets, that is, VMMRDB-495 [4] and our new DVMM. One notes that the network models are trained for the simpler vehicle make classification task compared to the vehicle model classification task, as seen from the number of vehicle classes given in Table 2. In Figure 5, we plot the accuracy of the make recogniser, the model recogniser and the corresponding 2B–2S during training process.

Table 6, Table 7 and Table 8 show the VMMR results on Compcar-51 [1], VMMRDB-51 [4], and new DVMM-51, respectively. Table 9 and Table 10 show the VMMR result on VMMRDB-495 [4] and new DVMM, respectively. Here, we study the performance of the model recogniser branch designed based on one of the following backbones: AlexNet, ResNet50, and DenseNet201. One can note that the vehicle model recogniser provides both the vehicle’s make and model, hence, for both 1B and 2B–2S solutions, the vehicle make classifications are also extracted from the vehicle model classification. Table 11 indicates the model complexity of 1B and 2B–2S by using different model recogniser backbones. In all tables, the best results at the vehicle model classification level are marked using bold font, while the best results at the vehicle make classification level are marked using underlines.

**Figure 6:**
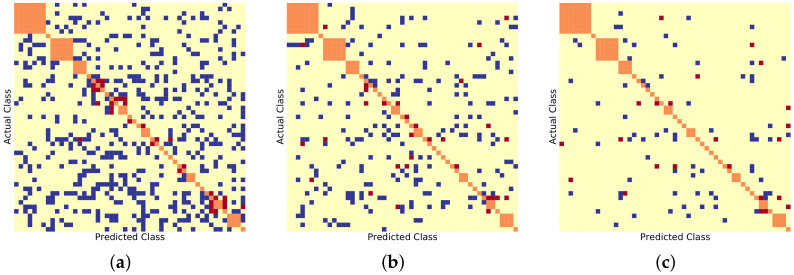
BDCM results on Compcar-51 [1] using different model recogniser backbones. Blue, yellow, and red dots mark the positions filled in with 1, 0, and −1, respectively. Orange dots mark the different vehicle models that have the same make. (**a**) AlexNet [15] backbone, (#blue, #yellow, #red) = (511, 1925, 32). (**b**) ResNet50 [16] backbone, (#blue, #yellow, #red) = (205, 2232, 31). (**c**) DenseNet201 [17] backbone, (#blue, #yellow, #red) = (71, 2374, 23).

**Figure 7:**
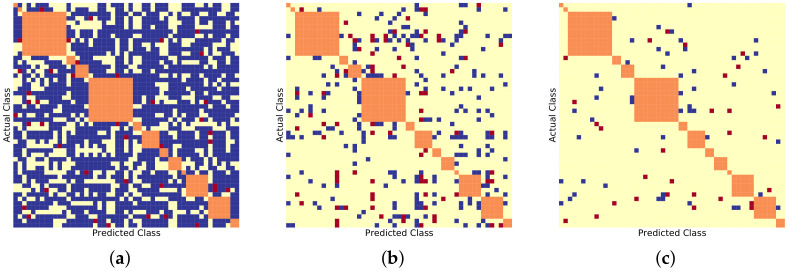
BDCM results on VMMRDB-51 [1] using different model recogniser backbones. Blue, yellow, and red dots mark the positions filled in with 1, 0, and −1, respectively. Orange dots mark the different vehicle models that have the same make. (**a**) AlexNet [15] backbone, (#blue, #yellow, #red) = (1523, 734, 41). (**b**) ResNet50 [16] backbone, (#blue, #yellow, #red) = (232, 1985, 81). (**c**) DenseNet201 [17] backbone, (#blue, #yellow, #red) = (52, 2217, 29).

**Figure 8:**
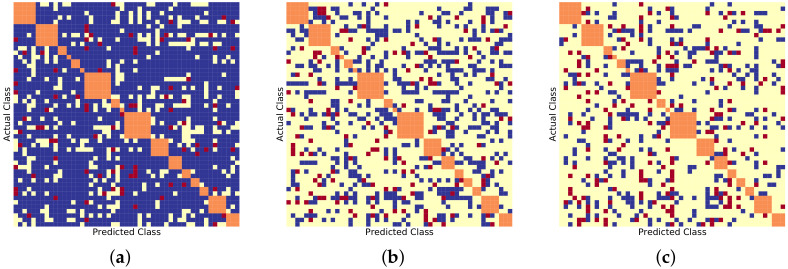
BDCM results on DVMM-51 using different model recogniser backbones. Blue, yellow, and red dots mark the positions filled in with 1,0, and −1, respectively. Orange dots mark the different vehicle models that have the same make. (**a**) AlexNet [15] backbone, (#blue, #yellow, #red) = (1871, 442, 89). (**b**) ResNet50 [16] backbone, (#blue, #yellow, #red) = (575, 1674, 153). (**c**) DenseNet201 [17] backbone, (#blue, #yellow, #red) = (356, 1848, 198).

**Figure 9:**
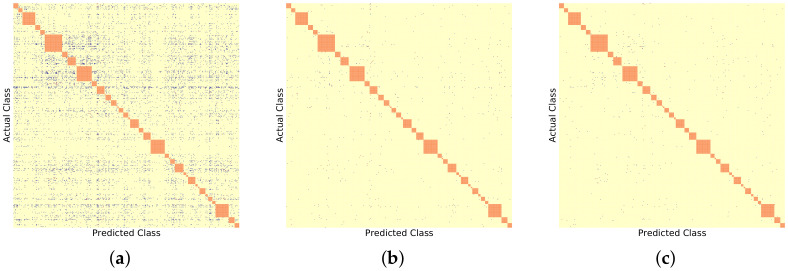
BDCM results on VMMRDB-495 [4] using different model recogniser backbones. Blue, yellow, and red dots mark the positions filled in with 1,0, and −1, respectively. Orange dots mark the different vehicle models that have the same make. (**a**) AlexNet [15] backbone, (#blue, #yellow, #red) = (9618, 225504, 734). (**b**) ResNet50 [16] backbone, (#blue, #yellow, #red) = (358, 235337, 161). (**c**) DenseNet201 [17] backbone, (#blue, #yellow, #red) = (273, 235450, 133).

**Figure 10:**
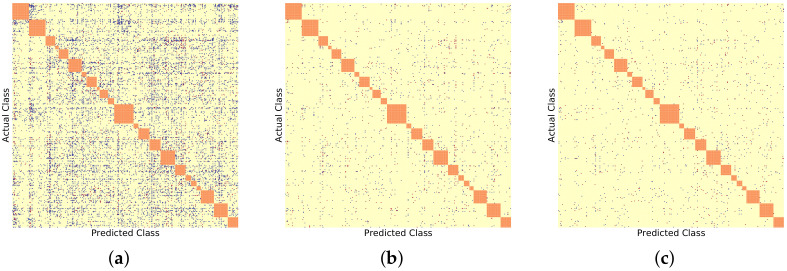
BDCM results on DVMM using different model recogniser backbones. Blue, yellow, and red dots mark the positions filled in with 1,0, and −1, respectively. Orange dots mark the different vehicle models that have the same make. (**a**) AlexNet [15] backbone, (#blue, #yellow, #red) = (9946, 89061, 1663). (**b**) ResNet50 [16] backbone, (#blue, #yellow, #red) = (1087, 98861, 722). (**c**) DenseNet201 [17] backbone, (#blue, #yellow, #red) = (774, 99318, 578).

Similarly, Figure 6, Figure 7 and Figure 8 show the BDCM visual results on the Compcar-51 [1], VMMRDB-51 [4], and new DVMM-51 dataset, respectively. Figure 9 and Figure 10 show the BDCM visual results on the VMMRDB-495 [4] and new DVMM datasets, respectively.

In Table 6, one can note that the proposed 2B–2S framework outperforms the corresponding 1B approach on the traditional Compcar-51 [1] dataset, for both vehicle make classification and vehicle model classification, and when employing any type of backbone for model recognition. Moreover, when the AlexNet backbone is employed, the make recognition accuracy based on the model recogniser is improved from 68.38% to 91.89%, the F1-score is also improved from 65.39% to 92.54%, and the *G*-score shows that the proposed 2B–2S framework reduces the classification confusion at both make and model recognising level, i.e., G(2B–2S)>G(1B). Similar results are achieved also when the ResNet50 [16] or DenseNet201 [17] backbone are employed. Overall, the best VMMR results on the dataset, 94.82% accuracy and 94.83% F1-score, are provided when both the make and model recogniser branches employ a DenseNet201 [17] backbone.

Figure 6 presents the visualization of the BDCM matrix, where one can note that the number of cases where 2B–2S provides a better result is much higher than the number of cases where 1B provides a better result, i.e., the number of blue dots is always larger than the number of red dots. This demonstrates the efficiency of the proposed 2B–2S framework, which introduces less inter-make ambiguity on the traditional Compcar-51 dataset. A similar conclusion can be drawn based on the VMMR results on the traditional VMMRDB-51 datset and the new DVMM-51 dataset, which, however, cover the same, but limited, number of vehicle models as Compcar-51.

In Table 7 and Table 8, one can note that the same improvement trend is obtained on the traditional VMMRDB-51 dataset and the new DVMM-51 dataset, irrespective of the type of backbone employed for model recognition. The results show that: (a) the make recognition accuracy based on the model recogniser is improved; (b) the F1-score of the proposed 2B–2S framework is larger than the F1-score of 1B; (c) the *G*-score shows that the inter-make ambiguity is highly decreased for the proposed 2B–2S framework.

Table 2 shows that both DVMM-51 and VMMRDB-51 datasets contain 10× more samples than Compcar-51. Since, for each vehicle model class, more samples are available for training, the neural networks provide better results on these datasets than over the Compcar-51 dataset. Figure 7 and Figure 8 present the visualization of the BDCM matrix, where one can note that the proposed 2B–2S framework continues to provide a better overall performance compared with 1B.

Table 9 shows the VMMR results on the traditional large-scale VMMRDB-495 [4] dataset. One can note that, based on the AlexNet [15] backbone, the accuracy of the model recogniser is improved from 60.69% to 72.26% and F1-score is improved from 53.63% to 66.87%. Similarly, the make recognition accuracy based on the model recogniser is improved with more the 25%. The ResNet50 [16] and DenseNet201 [17] backbones show the same improvement trend. We have similar observations for Table 10 reporting results on the new DVMM dataset.

Compared with the three small-scale *S*–51 datasets, both VMMRDB-495 and DVMM are much larger (around 20× larger than Compcar-51, around 1.5× larger than VMMRDB-51 and DVMM-51), and contains more than 6× more vehicle models.

One can note that VMMRDB-495 and DVMM are much more complex and more diverse than the *S*–51 datasets. Therefore, when working with such datasets, the VMMR problem becomes more complex as the network models are tested on a more diverse dataset. Nevertheless, one can note that the *G*-score results over such datasets (reported in Table 9 and Table 10) demonstrate that the proposed 2B–2S framework outperforms the corresponding 1B model recogniser and reduces the ambiguity at both make and model level classification.

**Figure 11:**
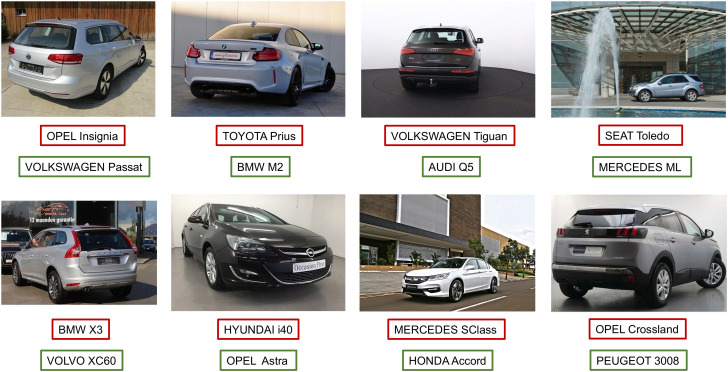
Visualization of the vehicle model classification confusion of 1B (red bounding boxes) and the corrected predictions of the corresponding 2B–2S (green bounding boxes).

In Figure 9 and Figure 10, the proposed 2B–2S framework continues to provide an overall better inter-make ambiguity reduction compared to 1B as the number of blue dots is always larger than the number of red dots. In Figure 11, we visualize the vehicle model confusion of 1B and the corresponding results of 2B–2S which are corrected by fusing the accurate make information from the make recogniser branch. Therefore, our proposed 2B–2S method is able to improve the performance of the VMMR task by significantly reducing the classification confusion.

One can observe that the proposed two-branch architecture is more complex compared to the single branch, as shown in Table 11 while with the increase of the dataset diversity, the difference in performance between 1B and 2B–2S is decreased; the latter observation inspires us future research on 2B–2S, as detailed in Section 6.

### 5.3. Ablation Study

In this section, we study the influence of the DM module in the proposed 2B–2S framework. We mainly focus on the following questions: *“Is it possible to further refine the output of the 1B model recogniser using the DM module?”* and *“Is the 2B–2S performance gain attributed to the two-branch approach or to the DM module?”*. Therefore, in this section, we introduce a new VMMR method, denoted 1B+DM, where the 1B model recogniser is further processed using the DM module.

Table 12 shows the VMMR results on the new DVMM-51 and DVMM datasets. The experiments are performed using the three model recogniser backbones, AlexNet, ResNet50, and DensNet201. The results of three VMMR solutions, that is, 1B, 1B+DM, and 2B–2S, are compared. The bold style fonts mark the best results for each dataset using different backbone configurations. One can note that the proposed 2B–2S framework always provides the best accuracy. However, 1B+DM provides better performance than 1B on DVMM using the AlexNet [15] and DenseNet201 [17] backbones, and worst performance in the other cases. Therefore, by simply inserting the DM module after the 1B model recogniser, the neural network does not always improve the model recognition performance. Moreover, the 2B–2S performance gain is mostly attributed to the proposed two-branch approach.

## 6. Discussion

The complex experimental evaluation presented in Section 5 demonstrates that the proposed 2B–2S framework based on the two-branch approach, with task-oriented branches for make and model recognition, can provide an outstanding performance when the neural network design is built based on complex backbones, like ResNet50 and DenseNet201. To reduce classification confusion, we proposed to follow a late feature fusion strategy by employing a DM module which further refines the vehicle model prediction by using the predicted vehicle make. This reduces the classification ambiguity at the expense of increased complexity. In future work, we will work on novel 2B–2S-based architectures that properly balance the trade-off between model complexity and robustness. To do so, the following research paths are interesting to be followed:

- Reduce model complexities by investigating the lightweight backbone architectures, like MobileNet-family [35] models, whose depthwise-pointwise convolution (DPC) pairs significantly decrease computational costs. We will study the potential of replacing the complex make and/or model branches partly or completely by the DPC structures of MobileNet.
- We can follow an early feature fusion strategy whereby intermediary multi-resolution features are extracted from the two backbones, followed by an advanced fusion module that integrates intermediary multi-resolution features from the make and model branches, similar to our research in [36,37].

## 7. Conclusions

The paper proposes a new large-scale vehicle make and model recognition dataset, DVMM, which covers the most popular vehicle makes and models available in the European automotive market. DVMM contains vehicle images with different colors, captured from different viewpoints and under various conditions, including camera types, image resolutions, illumination conditions and background settings. A novel deep-learning-based VMMR framework, 2B–2S, is proposed based on a two-branch processing and two-stage network training strategy. A novel metric is introduced to measure the ability to reduce classification confusion of the proposed 2B–2S architecture compared to the corresponding single-branch model recognition method. A thorough experimental evaluation using three well-known backbone architectures performed on small-, medium-, and large-scale VMMR datasets demonstrate that the proposed 2B–2S framework outperforms the corresponding one-branch approach, providing lower vehicle model confusion and significantly reducing the inter-make ambiguity.

## Figures and Tables

**Table 1:** Layer configuration of Decision Module.

Layer	Output Classes	Activation
Concatenate	#Make + #Model	-
Fully-connected	1024	ReLU
Fully-connected	1024	ReLU
Fully-connected	768	ReLU
Fully-connected	#Model	*Softmax*

**Table 2:** VMMR Dataset Specifications.

VMMR Dataset	Vehicle Classes			# of Image Samples	
	#Make	#Model		Train (70%)	Test (30%)
Compcar-51 [1]	23	51		6382	2736
VMMRDB-51 [4]	14	51		100,930	43,257
DVMM-51	17	51		87,750	37,608
VMMRDB-495 [4]	43	495		172,403	71,082
DVMM	23	326		159,924	68,539

**Table 3:** Detailed training schema on DVMM with different model recognisers.

Model Recogniser	Stage 1		Stage 2	
Backbone	Initial lr	Weight Factor	Initial lr	Weight Factor
AlexNet [15]	$10^{-5}$	0.1	$5\times 10^{-5}$	0.2
ResNet50 [16]	$10^{-4}$	0.1	$1\times 10^{-5}$	1.0
DenseNet201 [17]	$10^{-4}$	0.1	$5\times 10^{-5}$	0.2

**Table 4:** Make recognition results with different backbones over DVMM and DVMM-51.

Make Recogniser	Acc (%)	
Backbone	DVMM-51	DVMM
AlexNet [15]	69.82	75.68
ResNet50 [16]	92.02	90.62
DenseNet201 [17]	**96.82**	**93.89**

**Table 5:** Vehicle make recognition results for 1B using a DenseNet201 [17] backbone.

Metric	Training Dataset				
	Compcar-51 [1]	VMMRDB-51 [4]	DVMM-51	VMMRDB-495 [4]	DVMM
Acc (%)	98.94	97.27	96.82	96.73	93.89
F1 (%)	98.94	97.26	96.81	96.20	91.77

**Table 6:** VMMR results over Compcar-51 [1].

Method				1B			2B–2S	
Classification Level				Make	Model		Make	Model
Model Recogniser Backbone	AlexNet [15]	Acc (%)		68.38	63.24		91.89	**78.87**
		F1 (%)		65.39	62.69		92.54	**78.78**
		G (%)		0.16	0.92		99.84	**99.08**
	ResNet50 [16]	Acc (%)		90.39	84.54		98.90	**91.32**
		F1 (%)		89.38	84.46		98.82	**89.25**
		G (%)		2.06	24.23		97.94	**75.77**
	DenseNet201 [17]	Acc (%)		96.89	93.57		98.98	**94.82**
		F1 (%)		96.67	93.55		98.91	**94.83**
		G (%)		10.96	20.34		89.04	**79.66**

**Table 7:** VMMR results over VMMRDB-51 [4].

Method				1B			2B–2S	
Classification Level				Make	Model		Make	Model
Model Recogniser Backbone	AlexNet [15]	Acc (%)		70.09	61.58		97.27	**78.81**
		F1 (%)		62.94	59.56		96.52	**77.82**
		G (%)		1.93	2.49		98.07	**97.51**
	ResNet50 [16]	Acc (%)		96.42	93.40		97.05	**93.83**
		F1 (%)		95.34	93.33		96.23	**93.77**
		G (%)		17.42	23.75		82.58	**76.25**
	DenseNet201 [17]	Acc (%)		98.04	95.80		98.09	**95.85**
		F1 (%)		97.38	95.77		97.42	**95.82**
		G (%)		30.77	33.80		69.23	**66.20**

**Table 8:** VMMR results over DVMM-51.

Method				1B			2B–2S	
Classification Level				Make	Model		Make	Model
Model Recogniser Backbone	AlexNet [15]	Acc (%)		74.37	60.45		96.84	**73.58**
		F1 (%)		68.81	59.29		96.21	**73.65**
		G (%)		1.55	5.38		98.45	**94.62**
	ResNet [16]	Acc (%)		94.04	88.87		95.65	**89.90**
		F1 (%)		92.73	88.86		94.91	**89.89**
		G (%)		8.36	10.41		94.64	**89.59**
	DenseNet201 [17]	Acc (%)		96.73	93.67		97.24	**93.95**
		F1 (%)		96.03	93.67		96.72	**93.96**
		G (%)		21.21	26.75		78.79	**73.25**

**Table 9:** VMMR results on VMMRDB-495 [4].

Method				1B			2B–2S	
Classification Level				Make	Model		Make	Model
Model Recogniser Backbone	AlexNet [15]	Acc (%)		70.81	60.69		96.20	**72.26**
		F1 (%)		63.64	53.63		95.40	**66.87**
		G (%)		1.66	3.92		98.34	**96.08**
	ResNet50 [16]	Acc (%)		97.24	92.38		97.55	**92.55**
		F1 (%)		97.12	89.50		97.47	**89.74**
		G (%)		20.80	20.80		79.20	**67.24**
	DenseNet201 [17]	Acc (%)		98.03	93.60		98.24	**93.70**
		F1 (%)		97.92	91.04		98.16	**91.11**
		G (%)		23.25	34.89		76.75	**65.11**

**Table 10:** VMMR results on DVMM.

Method				1B			2B–2S	
Classification Level				Make	Model		Make	Model
Model Recogniser Backbone	AlexNet [15]	Acc (%)		75.56	62.65		92.82	**67.96**
		F1 (%)		67.93	51.36		90.45	**59.36**
		G (%)		3.99	10.48		96.01	**89.52**
	ResNet50 [16]	Acc (%)		95.10	89.73		95.71	**89.87**
		F1 (%)		93.93	**86.98**		94.65	86.95
		G (%)		26.01	40.98		73.99	**59.02**
	DenseNet201 [17]	Acc (%)		96.20	91.86		96.49	**91.90**
		F1 (%)		95.32	**89.78**		95.66	89.64
		G (%)		32.15	46.24		67.85	**53.76**

**Table 11:** Model complexity comparison with different model recognisers.

Model Recogniser	# of Param.	
Backbone	1-B	2B–2S
AlexNet [15]	60.3M	80.4M
ResNet50 [16]	24.2M	45.1M
DenseNet201 [17]	18.7M	39.8M

**Table 12:** Ablation study over DVMM and DVMM-51.

Model Recogniser	Acc (%)			
Backbone	Dataset	1B	1B+DM	2B–2S
AlexNet [15]	DVMM-51	60.45	60.39	**73.58**
	DVMM	62.65	62.78	**67.96**
ResNet50 [16]	DVMM-51	88.87	88.84	**89.89**
	DVMM	89.73	89.71	**89.78**
DenseNet201 [17]	DVMM-51	93.67	93.66	**93.95**
	DVMM	91.86	91.87	**91.90**

## Data Availability

Not applicable.
